# Automated T1 and T2 mapping segmentation on cardiovascular magnetic resonance imaging using deep learning

**DOI:** 10.3389/fcvm.2023.1147581

**Published:** 2023-07-07

**Authors:** András Kalapos, Liliána Szabó, Zsófia Dohy, Máté Kiss, Béla Merkely, Bálint Gyires-Tóth, Hajnalka Vágó

**Affiliations:** ^1^ Department of Telecommunications and Media Informatics, Faculty of Electrical Engineering and Informatics, Budapest University of Technology and Economics, Budapest, Hungary; ^2^ Semmelweis University, Heart and Vascular Centre, Budapest, Hungary; ^3^ Siemens Healthcare, Budapest, Hungary; ^4^ Department of Sports Medicine, Semmelweis University, Budapest, Hungary

**Keywords:** T1 and T2 mapping, MRI, cardiac segmentation, deep learning, artificial intelligence

## Abstract

**Introduction:**

Structural and functional heart abnormalities can be examined non-invasively with cardiac magnetic resonance imaging (CMR). Thanks to the development of MR devices, diagnostic scans can capture more and more relevant information about possible heart diseases. T1 and T2 mapping are such novel technology, providing tissue specific information even without the administration of contrast material. Artificial intelligence solutions based on deep learning have demonstrated state-of-the-art results in many application areas, including medical imaging. More specifically, automated tools applied at cine sequences have revolutionized volumetric CMR reporting in the past five years. Applying deep learning models to T1 and T2 mapping images can similarly improve the efficiency of post-processing pipelines and consequently facilitate diagnostic processes.

**Methods:**

In this paper, we introduce a deep learning model for myocardium segmentation trained on over 7,000 raw CMR images from 262 subjects of heterogeneous disease etiology. The data were labeled by three experts. As part of the evaluation, Dice score and Hausdorff distance among experts is calculated, and the expert consensus is compared with the model’s predictions.

**Results:**

Our deep learning method achieves 86% mean Dice score, while contours provided by three experts on the same data show 90% mean Dice score. The method’s accuracy is consistent across epicardial and endocardial contours, and on basal, midventricular slices, with only 5% lower results on apical slices, which are often challenging even for experts.

**Conclusions:**

We trained and evaluated a deep learning based segmentation model on 262 heterogeneous CMR cases. Applying deep neural networks to T1 and T2 mapping could similarly improve diagnostic practices. Using the fine details of T1 and T2 mapping images and high-quality labels, the objective of this research is to approach human segmentation accuracy with deep learning.

## 1. Introduction

Cardiovascular diseases are the leading cause of death worldwide, claiming approximately 18 million lives a year (1). Its high morbidity burden further underscores the need for improving our methods to address these diseases. Recent advances in cardiovascular imaging has enhanced our capability to better understand, prevent, diagnose and stratify patients (2).

Cardiovascular Magnetic Resonance (CMR) is the gold standard imaging method for cardiac anatomy and function assessment, as well as enabling non-invasive tissue characterization. Besides late gadolinium enhancement, parametric cardiac mapping is a widely used technique to quantitatively measure the properties of the myocardial tissue. It provides direct visualization of tissue-specific MR properties like T1, T2 and T2*. Parametric mapping provides pixel-by-pixel representations of the numerical T1 and T2 myocardial tissue properties defined in units of time (milliseconds). In recent years mapping values have been established as valuable descriptors of myocardial alterations (3). These variations reflect on specific intracellular and/or extracellular tissue changes linked to specific pathologies. For example, intracellular accumulation of glycosphingolipid in Anderson-Fabry disease, extracellular fibrosis in cardiac amyloidosis or extra- and intracellular oedema in acute myocardial inflammation or injury.

Currently, the 2020 Society of Cardiovascular Magnetic Resonance (SCMR) guideline recommends using manually drawn regions of interest (ROI) within the mid-ventricular or basal septal segment to assess the global tissue character (4). This contouring practice reduces variability caused by motion artifacts, primarily affecting the lateral wall. On the other hand, manual ROI segmentation enables reader subjectivity and might increase interobserver variability. Moreover, several pathological alterations disproportionately affect the lateral wall of the left ventricle (LV), such as myocardial injury caused by acute myocarditis, necessitating reliable tissue quantification in all segments of the left ventricular (LV) myocardium (5). In contemporary clinical practice, labor-intensive manual segmentation is the prerequisite to extracting key clinical measures of the tissue character, such as global and segmental T1 and T2 mapping values. This task is not only time-consuming but also prone to several subjective practices, preventing the generalization of the results.

In recent years artificial intelligence driven methods have transformed the segmentation and reporting practices in CMR imaging. This effort, motivated by the time-consuming manual contouring for precise quantification of volumes- and function, has led to a multitude of automated tools for cine movie segmentation (6). In contrast, reliable tools for automated mapping contour generation is still scarce and did not reach clinical applicability.

In general, segmentation tools developed within the frameworks of large, generally very healthy cohorts such as the UK Biobank (UKB) Imaging substudy are mainly exposed to healthy anatomy (7); therefore, the performance of these algorithms is lower within clinical cohorts. Tools developed within these cohort studies are further limited by the specific set of acquired slices. For example the UKB contains only one mid ventricular T1 mapping slice per participant, which prevents algorithm training in hard-to-learn basal or apical slices. Moreover, a special type of mapping imaging sequence was used, which is not widespread in the clinical routine, potentially limiting clinical implementation of such algorithms.

In this study, we aimed to develop an automated segmentation tool for mapping images within a diverse clinical cohort to promote the generalizability of automated mapping segmentation tools.

## 2. Related work

There are several CMR mapping sequences suitable for tissue quantification. The most common variants based on inversion-recovery, saturation recovery or the combination of both (3). In this work, we are focusing on the most commonly used T1 mapping sequence in the clinical routine: the modified Look-Locker inversion recovery (MOLLI) (8) which permits measurement of T1 times in a single breath hold fashion over 17 heart beats.

Deep learning (DL) has become one of the most widely used approaches in cardiac imaging in several imaging modalities (e.g., Magnetic Resonance Imaging, X-Ray, Computed Tomography, Ultrasound). These modalities enable non-invasive qualitative and quantitative assessment of anatomical structures, functions and support the radiologist/cardiologist for diagnosis, follow-up, and prognosis as well.

In general DL, can be used within two fields of CMR, a.) at k-space field, when machine learning reconstruction approaches were used to learn non-linear optimization and improve the CMR reconstruction when an MR-based acceleration technique (e.g., Compressed Sensing) was used (9). In another perspective DL can be applied at k-space to suppress the artifacts before the image reconstruction (10).

The second field, b.) when DL could be useful is the image domain. A huge number of studies concentrate on cardiac MRI segmentation, because those provide an efficient way for segmenting tissues (e.g., left ventricle, right ventricle, vessels, etc.).

Tran (11) applies DL to segment the left ventricle, right ventricle and myocardium on short-axis images. Khened et al. (12) applied a U-net for segmentation with large anatomical variability. The majority of ventricle and myocardial segmentation studies used 2D networks instead of 3D networks, due to the low resolution, motion artifacts and the limited availability of 3D dataset. Fully Convolutional Neural Networks (FCNs) could be useful at atrial segmentation (13), scar segmentation/classification (14) or whole heart segmentation (15). Hybrid segmentation is also worth mentioning. In this case the DL-based algorithm is combined with the traditional segmentation approaches (e.g. deformable models (16) or atlas-based methods (17). These algorithms provide the segmentation accuracy. Chen et al. (6) give a very comprehensive overview about the DL-based applications, their summary illustrates that to date the majority of DL segmentation tools have focused on cine SAX and late gadolinium enhanced images providing limited examples of automated mapping segmentations tools. Recently, Evan Hahn et al. used DL to perform segmentation on T1 mapping images (7), they achieved high performance, nevertheless ShMOLLI sequences were used, which have a very limited availability. Rui Guo also applied deep learning to accelerate cardiac T1 mapping images with inline MyoMapNet (18).

## 3. Materials and methods

In this chapter, we introduce the data we utilize in this study, its acquisition method, preprocessing and annotation methodology. Then we present a deep convolutional network based automated segmentation method, including the network’s architecture and its training.

### 3.1. Experimental data: participants

Overall, we included 262 participants in our study (71% male, mean age 46±16 years). To permit the consideration of a wide variety of cardiovascular phenotypes we consecutively included all clinical patients who were referred to CMR examination between September and November 2021 at Semmelweis University Heart and Vascular Center. Furthermore, we randomly selected healthy volunteers from the Center’s CMR database.

**Figure 1:**
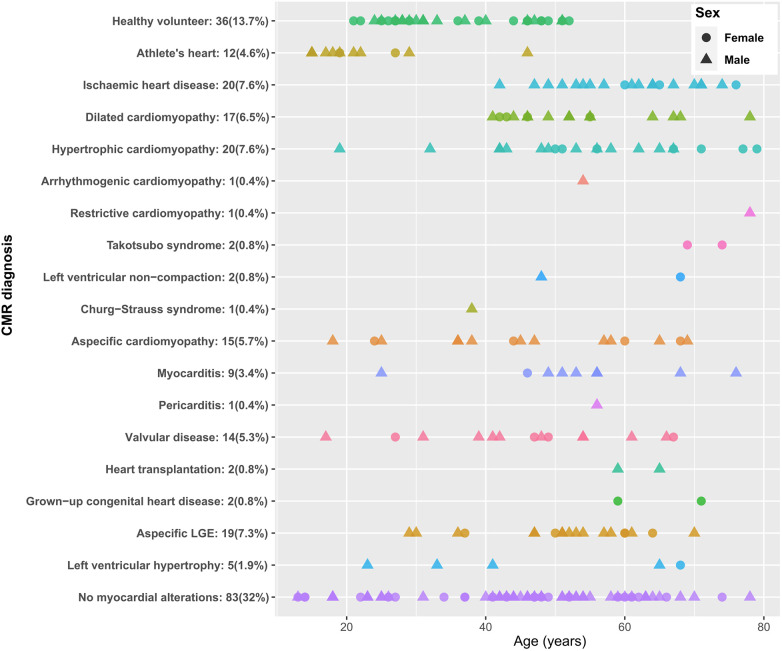
The proportion of healthy volunteers, athletes and pathologies in our dataset was reported using cardiovascular magnetic resonance (CMR) imaging. “No myocardial alterations” were reported, when the patient was referred to CMR due to a specific diagnostic question, but no pathology was found.

Diagnostic labeling was provided for each case by two independent, experienced CMR readers with more than four and five years of experience (EACVI exams completed). In case of uncertainty a third, EACVI level III reader was contacted for consensus. We labeled each participant as per the current clinical guidelines and expert recommendations. Please note, that this dataset is curated to produce a fair representation of the diversity of clinical datasets and to provide a real-life segmentation scenario for algorithm training. If the results of the CMR examination were not consistent with any specific underlying disease we provided the phenotypic label best describing the scan, including left ventricular hypertrophy, aspecific gadolinium enhancement, aspecific cardiomyopathy indicating further assessment. This approach will allow for the appreciation of the diversity of the data. For simplicity, we pulled together valvular diseases into one diagnostic label, as mitral prolapse, aortic stenosis and aortic insufficiency are often present at the same time. Diagnosis groups are shown in Figure 1.

### 3.2. Experimental data: image acquisition and preprocessing

CMR examinations were performed on a 1.5T MR scanner (Magnetom Aera, Siemens Healthcare, Erlangen, Germany) using a spine and an 18-channels phased array coil. The development and testing data contain 638 CMR pre-contrast (native) T1 maps where the typical imaging parameters are: TR=2.7 ms; TE=1.12 ms; \,pixel size=1.5×1.5 mm; slice thickness=8 mm; $\text{\textbackslash{},flip angle}=35^{\circ }$; FOV=323×360; and 720 T2-maps with the following parameters: TR=2.4 ms; TE=1.06 ms; \,pixel size=1.5×1.5 mm; slice thickness=8 mm; $\text{\textbackslash{},flip angle}=70^{\circ }$; FOV=315×360. MOLLI technique was used at both maps. All images were short-axis views of the left ventricular (LV) myocardium. We acquired mapping sequences in three short-axis slices according to the anatomical landmarks: in the basal, midventricular and apical part of the heart. Two-fold accelerated parallel imaging technique (GRAPPA) was used to shorten the breath-hold.

Conventional MOLLI sequence was used to produce T1 and T2 mapping images as output maps from individual images ascertained at predefined intervals. T2 maps were generated by using a T2-prepared steady-state free precession sequence (19) with three different echo times (TE): ${\mathrm{TE}}^{1}=0$ ms, ${\mathrm{TE}}^{2}=25$ ms, ${\mathrm{TE}}^{3}=55$ ms (20). In order to generate T2 maps, several images (in our case 3) with varying T2 sensitivity are acquired at the same cardiac phase over multiple cardiac cycles. Then, a mono-exponential curve is fit at each pixel on the T2-weighted images and finally a T2 map is generated (20). For T1 maps generation, multiple (in our case 8) T1 weighted images are acquired at different times after the inversion pulse and then the data can be fit to an equation: A−Bexp⁡(−t/T1), where A and B are fitting parameters, t is the time after the preparation pulse and T1 is the T1 relaxation time (21). To increase the training and validation sample of our algorithm we included each image individually as well as T1 and T2 parametric maps.

We split the dataset into three partitions: training, validation and testing with 65%-20%-15% ratios of all study participants respectively. This partitioning is fixed for all trainings to help reproducibility and comparability of models. We perform partitioning on a case level (instead of for images) to avoid any information from the test set being included in the train and validation partitions. I.e., all data of a patient is strictly included in only one of the training, validation, or test partitions. This partitioning results in 832, 287, 239 T1 and T2 maps (172, 50, 40 cases) in the training, validation and test sets respectively. A summary of the number of samples per slice in both the training and test sets can be found in Table 1.

**Table 1:** Number of mapping images for each slice in our training and test datasets.

	Basal	Midventricular	Apical	Total
Train	294	293	245	832
Test	80	80	79	239

### 3.3. Experimental data: ground truth segmentation, and interobserver subset

Ground truth segmentation was performed by two experienced observers using the Medis Suite Software (Medis Medical Imaging Software, The Netherlands). We draw the endo- and epicardial contours manually on the motion-corrected images. After visual quality control, endo- and epicardial contours were manually drawn in basal, midventricular and apical slices covering the LV myocardium. To minimize partial volume effect care was taken to exclude the border zones between myocardium and the blood pool during segmentation.

In the test set (15% of the complete dataset, n=40 patients), we evaluate the interobserver variability of the manual segmentation approach. Three readers with one, five and four years of experience in CMR reporting, respectively provided the segmentation. They were blinded to clinical label and demographic characteristics of the patients.

### 3.4. Implementation: model architecture and training details

We apply a widely used U-Net (22) segmentation network to the cardiac mapping segmentation problem. Our motivation for choosing this architecture is to provide a method that can be trained quickly and achieve a strong baseline for segmentation. To simplify and shorten the training of this network we initialize the U-Net’s encoder with weights which were previously pretrained on a large and diverse general image dataset, ImageNet (23). Even though properties and distribution of data from the ImageNet dataset differ from MRI images, such transfer-learning approach is often used successfully in medical image analysis problems (24). As opposed to the encoder, we do not initialize the decoder from pretrained weights, instead train its weights for the mapping segmentation problem from random initialization.

**Figure 2:**
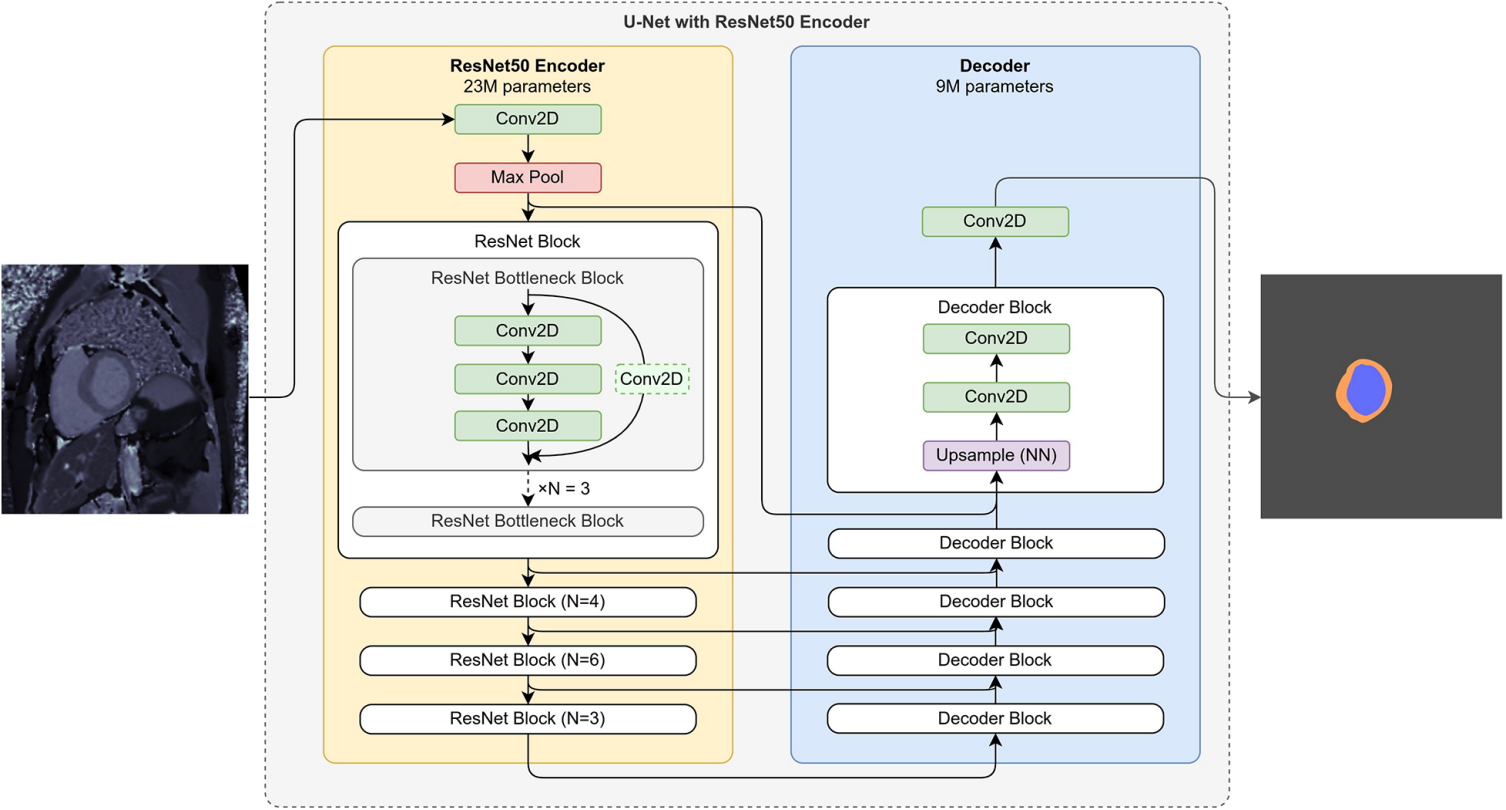
Deep neural network architecture: U-Net with ResNet-based encoder and convolutional decoder.

A U-Net-like encoder-decoder model can be constructed of multiple encoder and decoder architectures. Pretrained weights are available for many encoder architectures. We opted for a ResNet-50 (25) encoder due to its frequent use in computer vision literature for segmentation tasks. The encoder and the decoder consist of convolutional, pooling and upsampling layers, with 50 and 8 convolutional layers in the encoder and the decoder, respectively. The reason for choosing a smaller decoder is to introduce less randomly initialized weights into the segmentation network, thereby reducing its training time. The detailed architecture of our model is shown on Figure 2.

Our experts provide ground truth segmentation labelling as epi- and endocardial contours. These are represented as ordered two-dimensional point sets described by {(x,y)∈R} pixel coordinates for epicardial and endocardial left ventricle contours. Although this representation is more accurate and efficient than segmentation masks, the latter is required in order to train neural networks for segmentation. We convert contour point sets to segmentation masks, i.e., pixel-level classification labels, with three classes: left ventricular cavity (area enclosed by the endocardial contour), left ventricular myocardium (area between the endo- and epicardial contour), and “background,” which is the class assigned for pixels that do not belong to the first two classes, i.e. all pixels outside the epicardial contour. Finally, we generate segmentation masks to match the resolution of the input samples (thereby achieving pixel-level classification). We apply random augmentations to the input samples during training. These are cropping, resizing, contrast and intensity perturbations. We use fixed cropping and resizing to match the input size of the network during inference (which is 224×224 pixels).

**Figure 3:**
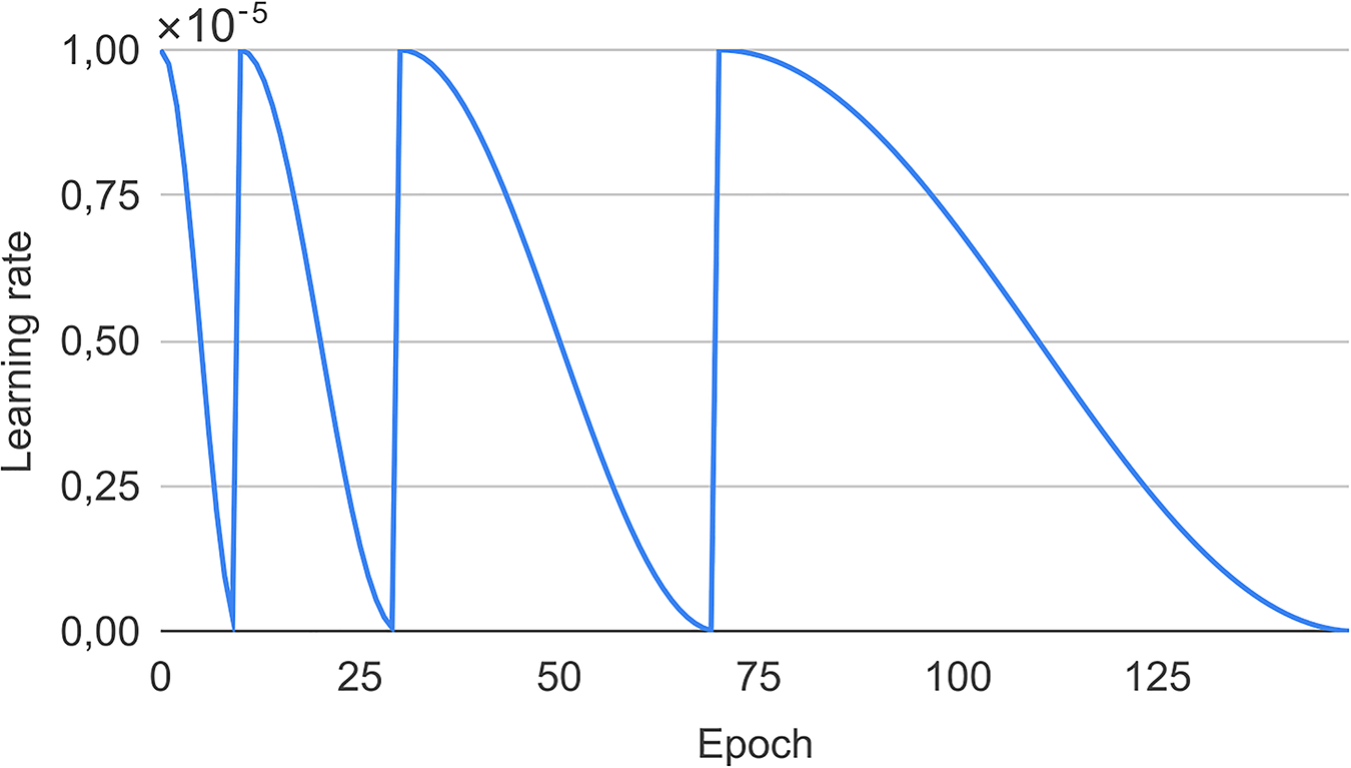
Learning rate schedule used throughout the training as proposed by Loshchilov and Hutter (28).

We train the segmentation network using the Adam gradient-based optimization algorithm (26). The objective function for segmentation training is the Jaccard loss (27). We train the network for 150 epochs, which takes 50–60 min on a single NVIDIA TESLA V100 GPU. Based on empirical analysis, we apply a periodic cosine annealing learning rate schedule (see Figure 3).

During the first 70 epochs we include both T1 and T2 parametric maps and the images used to construct them in the training sample set, however, during the last 80 epochs we limit the inputs to the T1, T2 parametric maps only. The first part of the training aims to achieve robust and well-generalizing performance by training on as many samples as possible, while the second half of the training aims to fine-tune the network to mapping segmentation. (The reason for the uneven 70–80 split of the epochs is to match the periodicity of the learning rate schedule.)

### 3.5. Implementation: hardware and software architecture

For training our automated segmentation method, we used a single Nvidia V100 16 GB GPU, 10 Intel(R) Xeon(R) Silver 4114 CPU cores (2.20 GHz) and 39 GB RAM. We implement our model and training using PyTorch Lightning and utilize methods from multiple open source repositories.^1^^,^^2^^,^^3^^,^^4^

Training our deep learning model on this system takes cca. 50–60 min, while inference on 100 images takes 12.3 sec. We use the same hardware for inference on the test set.

### 3.6. Evaluation metrics

This chapter presents the metrics and comparison baselines we used to evaluate the proposed segmentation method.

We evaluate the performance of our segmentation method based on two metrics, Dice score and Hausdorff distance (DH) computed between corresponding predicted contours and expert annotations. For a single sample with contours from multiple annotators we compare the same prediction to each expert’s contour. For computing the Dice score we convert ground truth contours to segmentation masks and compare these against predicted segmentation masks. Computing the Hausdorff distance requires predicted contours, which we obtain by fitting an epi- and endocardial contour on the predicted segmentation mask. We explain the applied contour fitting pipeline in the next paragraph. In addition to these metrics, we calculate myocardial T1 and T2 mapping values based on the contours predicted by our model and compare to those derived from expert-determined contours.

**Figure 4:**
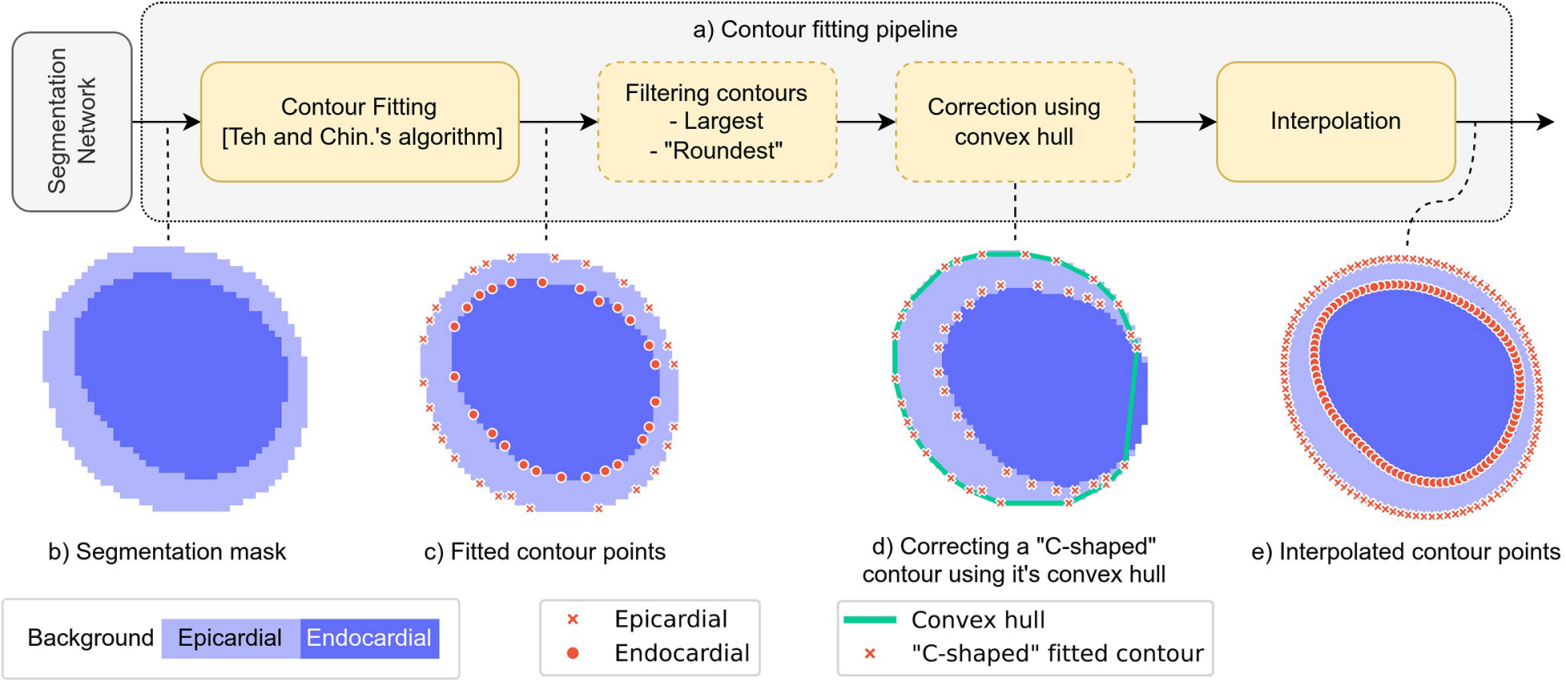
(**A**) Illustration of our algorithm that fits epi- and endocardial contours to segmentation masks. (**B**) Segmentation mask with the same resolution as the input image. (**C**) Contour points fitted on the low-resolution segmentation mask using a dominant point detection algorithm by C-H Teh and Roland T Chin (29). (**D**) Illustration of a case where correcting the contour using its convex hull improves the validity and accuracy of the contour. (**E**) Result the interpolation step which is performed by first fitting a smooth spline on the fitted contour points (30), then sampling it between the initial points. This step improves the numeric accuracy of contour-based metric computation (e.g. Hausdorff distance) and also enforces a smooth property on the fitted contours, similarly as the ones of expert annotators. On subfigure (A), steps indicated with dashed outline are only performed if necessary: - Filtering of contours is only necessary if the segmentation network incorrectly predicts multiple disjoint epi- or endocardial regions. We select the contour with the largest area and an approximately round shape (have a less than 1:3 with-height ratio) - Correcting a contour using its convex hull is necessary only in rare cases, when the segmentation mask includes a C-shaped region (e.g. the light blue region on (D)) In such case he fitted contour will inherit this C-shaped topology (see the red crosses on the figure). We can simply improve such contour by replacing in with its convex hull (green line on subfigure D)).

For fitting contours on predicted segmentation masks, we rely on an algorithm by Teh and Chin (29). However, to acquire accurate and robust contours, we apply further processing steps, as illustrated on Figure 4. Inaccurate predictions can include multiple distinct regions of the same class, which produce multiple epi- or endocardial contours on a single mapping. From these we select the ones which are “not too elongated” (have a less than 1:3 with-height ratio) and select the one with the largest area. On some mapping images the myocardium appears very thin, resulting in C shaped endocardial contours, which we correct by replacing the contour with its convex hull. Note, that we only perform this replacement if the contour is not fully closed (“C-shaped”), which we determine based on the difference between the contour’s and its convex hull’s area. As a last step, we interpolate the fitted contour with a B-spline (30) to improve the accuracy of the Hausdorff distance and Mean Surface Distance metric computation.

### 3.7. Implementation: experiment design

We compute evaluation metrics on T1 and T2 mapping images of our test set for which contours are available from 3 expert annotators. The train and the test partitions are disjunct sets. As our aim is to develop a segmentation method for T1 and T2 parametric maps, we only use these during evaluation, while during training we also use images that were used to compute the parametric maps. We compute metrics on each slice and mapping type (T1 or T2) separately and report the distributions and cumulate results of these.

In order to evaluate the statistical significance of differences in segmentation metrics of our model and the agreement of experts, we employed one-way Analysis of Variance (ANOVA) with Welch’s correction and the Tukey Post-Hoc Test, considering a significance level of p<0.05. We used Kruskal-Wallis non-parametric one-way ANOVA test to evaluate the statistical significance of differences in T1 and T2 values and used Dwass-Steel-Critchlow-Fligner test for post-hoc pairwise comparisons. We indicate the results of these tests in Tables 2 and 3 to 5 with identical letters in superscript for results that are not significantly different. We used the jamovi statistical software for these tests (31).

**Table 2:** Mean metrics for comparing our method to expert annotators.

Mean metrics	Automated VS expert annotation	Agreement of three experts (interobserver)
IoU - Jaccard index ⇑ (%)	80.02${}^{a}$	85.22${}^{b}$
Dice score ⇑ (%)	86.40${}^{a}$	90.81${}^{b}$
Hausdorff distance ⇓ (mm)	4.68${}^{a}$	2.65${}^{b}$
Mean surface distance ⇓ (mm)	2.10${}^{a}$	1.12${}^{b}$

For each metric, differences are statistically significant (p<0.001, also denoted with different lettering in superscripts). ⇑ indicates higher-the-better metrics, while metrics ⇓ are lower-the-better.

**Table 3:** Mean metrics for comparing our method to expert annotators, displayed separately for T1 and T2 mapping images.

	Automated vs expert annotation		Agreement of three experts (interobserver)	
	T1	T2	T1	T2
IoU - Jaccard index ⇑ (%)	81.7${}^{a}$	78.39${}^{\alpha }$	84.14${}^{b}$	86.25${}^{\beta }$
Dice score ⇑ (%)	87.8${}^{a}$	85.02${}^{\alpha }$	89.84${}^{b}$	91.73${}^{\beta }$
Hausdorff distance ⇓ (mm)	3.97${}^{a}$	5.39${}^{\alpha }$	2.89${}^{b}$	2.41${}^{\beta }$
Mean surface distance ⇓ (mm)	1.77${}^{a}$	2.44${}^{\alpha }$	1.34${}^{b}$	1.00${}^{\beta }$
Number of training samples	378	454	—	—
Number of test samples	116	119	116	119

For each metric and mapping type (T1 or T2), differences are statistically significant (p<0.001, also denoted with different lettering in superscripts). ⇑ indicates higher-the-better metrics, while metrics ⇓ are lower-the-better.

**Table 4:** Mean metrics for comparing our method to expert annotators, displayed separately for each slice.

	Automated vs expert annotation			Agreement of three experts (interobserver)		
	Basal	Midventricular	Apical	Basal	Midventricular	Apical
IoU - Jaccard index ⇑ (%)	84.06${}^{a}$	81.83${}^{\alpha }$	73.13${}^{A}$	87.5${}^{b}$	85.49${}^{\beta }$	81.78${}^{B}$
Dice score⇑ (%)	90.09${}^{a}$	88.24${}^{\alpha }$	79.86${}^{A}$	92.62${}^{b}$	90.98${}^{\beta }$	88.12${}^{B}$
Hausdorff distance ⇓ (mm)	3.59${}^{a}$	3.52${}^{\alpha }$	7.41${}^{A}$	2.51${}^{b}$	2.6${}^{\beta }$	2.91${}^{B}$
Mean surface distance ⇓ (mm)	1.57${}^{a}$	1.59${}^{\alpha }$	3.37${}^{A}$	1.05${}^{b}$	1.12${}^{\beta }$	1.39${}^{B}$

For each metric and each slice, differences are statistically significant (p<0.001, also denoted with different lettering in superscripts). ⇑ indicates higher-the-better metrics, while metrics ⇓ are lower-the-better.

**Table 5:** Median T1 & T2 values computed on our model’s output and for each observer.

	Model	Observer 1	Observer 2	Observer 3
T1 (ms)	1041.7 (*110.5*)${}^{a}$	1017.4 (*62.3*)${}^{a}$	1025.9 (*69.7*)${}^{a}$	1032.9 (*76.17*)${}^{a}$
T2 (ms)	56.2 (*13.2*)${}^{a}$	49.5 (*5.3*)${}^{b}$	51.6 (*6.8*)${}^{c}$	51.7 (*6.4*)${}^{c}$

Inter Quartile Range (IQR) is also reported in brackets. For each observer the median and IQR are computed over cca. 113 samples. For each mapping value, medians with different letters in superscript differ significantly (p<0.05).

## 4. Results

### 4.1. General segmentation accuracy

**Figure 5:**
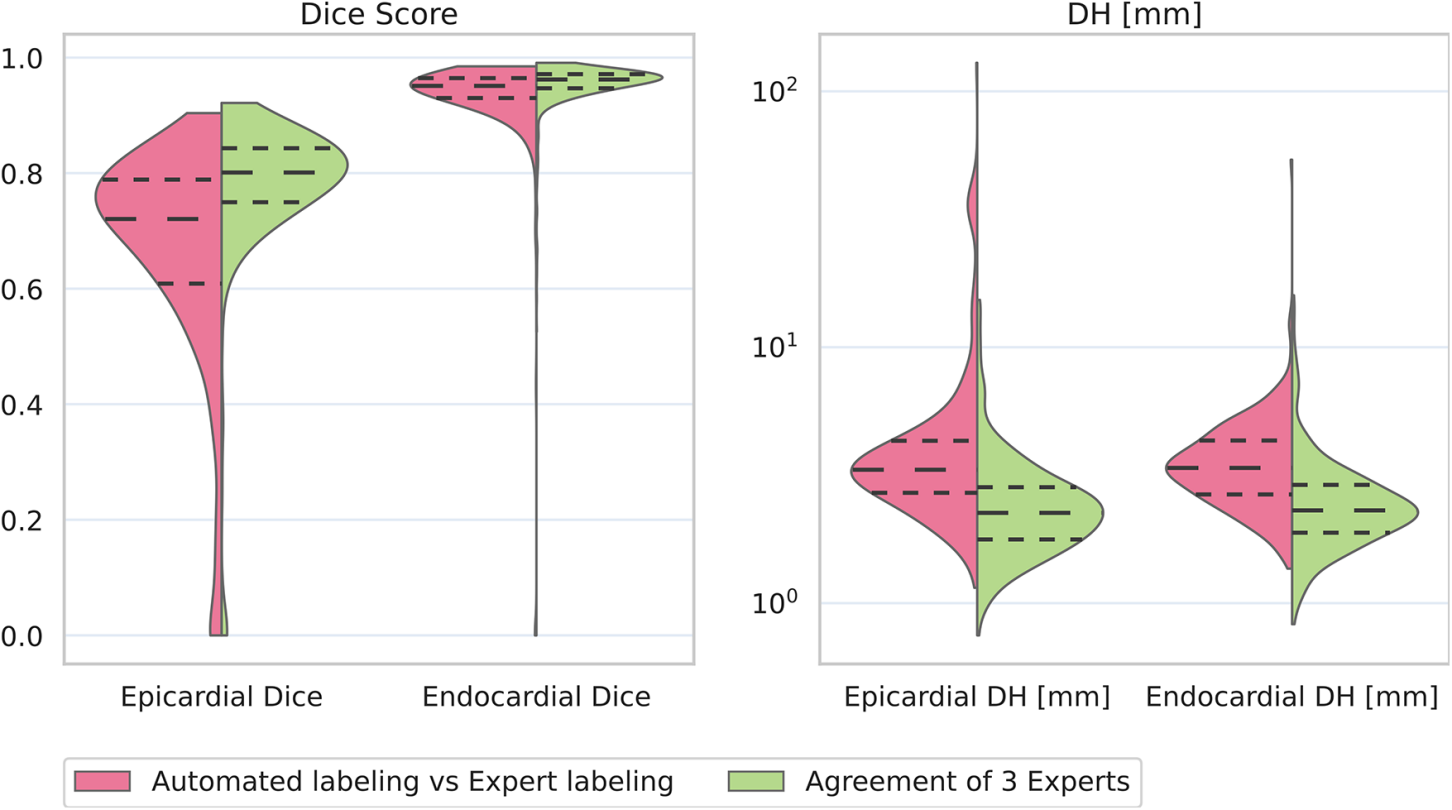
Violin plots showing the results of pairwise comparison of our automated method (pink halves) to expert annotators (green halves). Horizontal dashed lines show median and Q1, Q3 quartiles. Violin plots are cut at the bounds of underlying data. Note the logarithmic y-axis on the Hausdorff Distance. We plot epi- and endocardial results separately to allow for accurate reporting of Dice score results (plotting the mean of epi- and endocardial dice score would hide the difference in these two metrics).

Figure 5 and Table 2 show our primary results. On Figure 5, both metrics on epi- and endocardial contours indicate that our automated segmentation method performs comparable to how expert annotators agree, as the distributions and even standard deviation bands overlap. The Dice score and IoU score both show 5% difference between our model and experts’ agreement (see Table 2).

The similar epi- and endocardial Hausdorff distance distribution of expert’s agreement results indicates that experts can label epi- and endocardial contours equally accurately. This observation is in-line with clinical experience. In contrast, the epicardial Dice score shows approximately 15% lower agreement between experts compared to endocardial results. This can be explained by the difference in how we compute epi- and endocardial Dice score. We compute the epicardial Dice score on the myocardium, which is a region with ring-like topology, while the endocardial Dice is computed on the closed disk-like region of the left ventricle. The sensitivity of the Dice score to similar errors on ring-like regions is higher than on closed disks, which can explain the lower epicardial Dice results. Therefore, we argue that our automated tool performs comparable on epicardial contours as well.

In Table 3, we present our results separately for T1 and T2 mapping images. All metrics indicate that our model performs better on T1 mapping than on T2 mapping, while metrics for experts indicate greater agreement on T2 mapping. Based on our comparison of expert agreements with our model, we observe that the model is closest to expert accuracy on T1 mapping, while the gap between model and expert accuracy is greater on T2 mapping. It can be explained by the fact that T1 mapping images are often noisier than T2 mapping images, which makes it more difficult for experts to determine epi- and endocardial contours with accuracy, but our method can learn to invariantly locate them despite these noises.

### 4.2. Analysis of segmentation accuracy at basal, midventricular and apical short-axis slices

**Figure 6:**
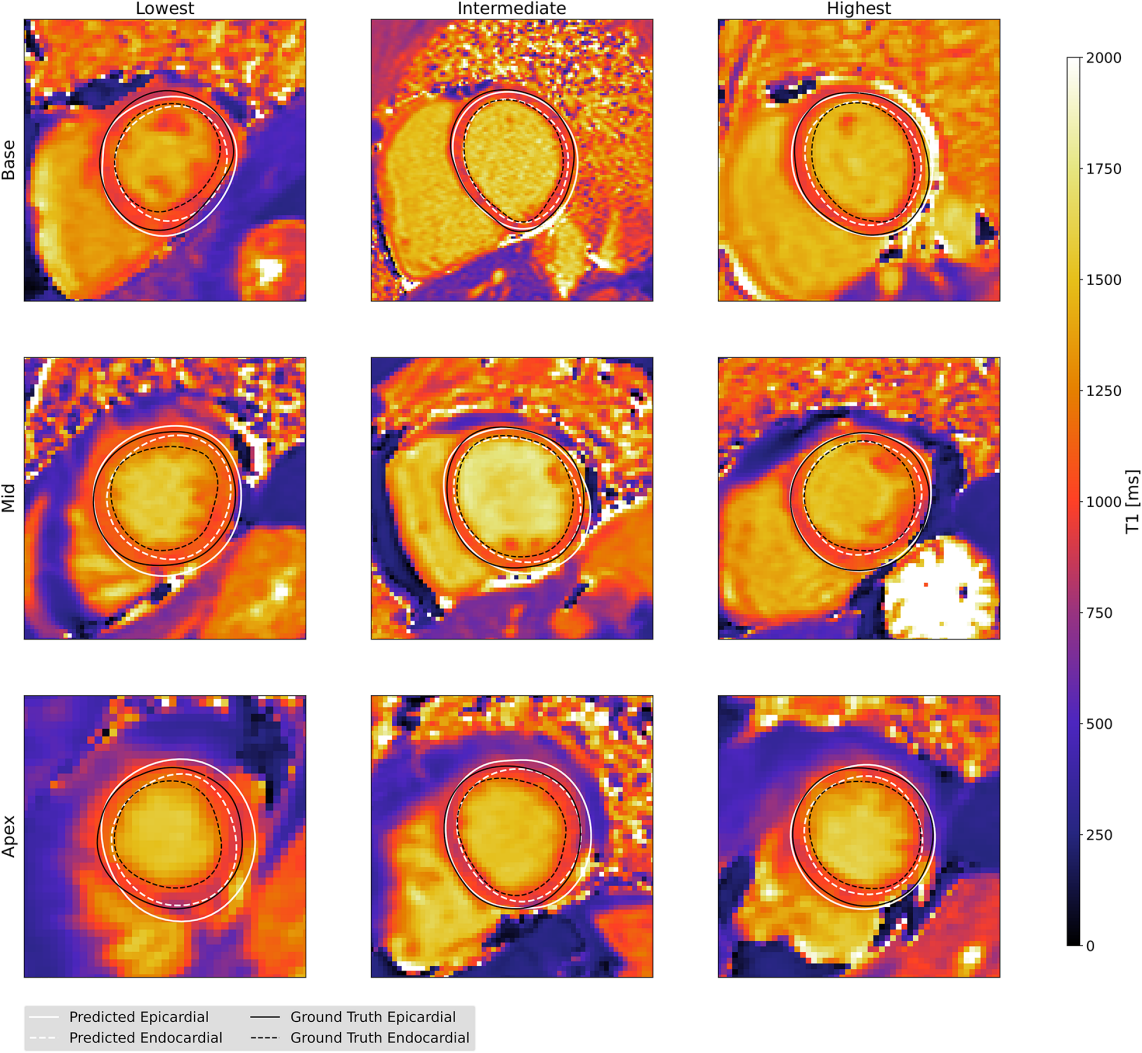
Predicted and ground truth contours with low, intermediate and high Hausdorff distance results, selected separately for apical, midventricular and basal T1 maping images.

**Figure 7:**
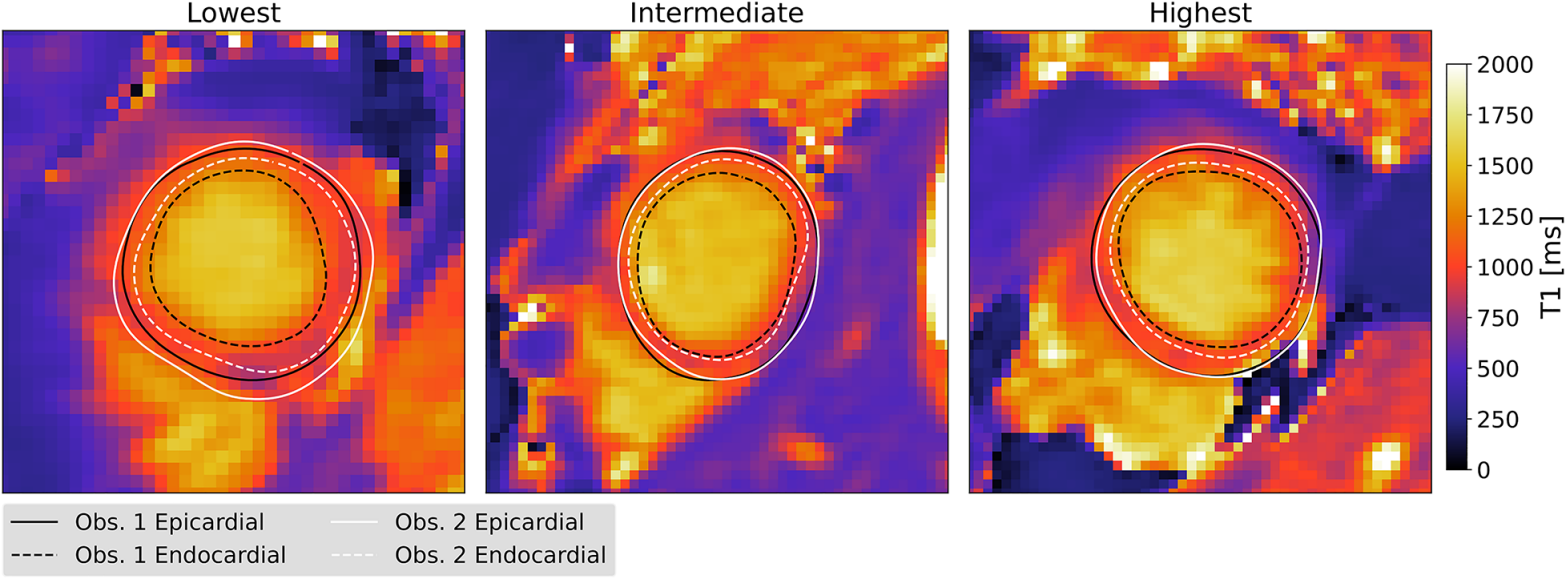
Example contours from two experts with different levels of agreement (measured by Hausdorff distance), plotted for two observers as a reference. All subfigures show apical T1 maps.

Figure 6 illustrates qualitative results for our automated method and allows for comparison to contours identified by an expert. The three columns show examples with lowest, intermediate, and highest agreement between an expert and our model, separately for each slice. The rows of Figure 6 show examples for apical, midventricular and basal slices respectively. We include Figure 7 to illustrate the different levels of agreement between two experts as a reference.

**Figure 8:**
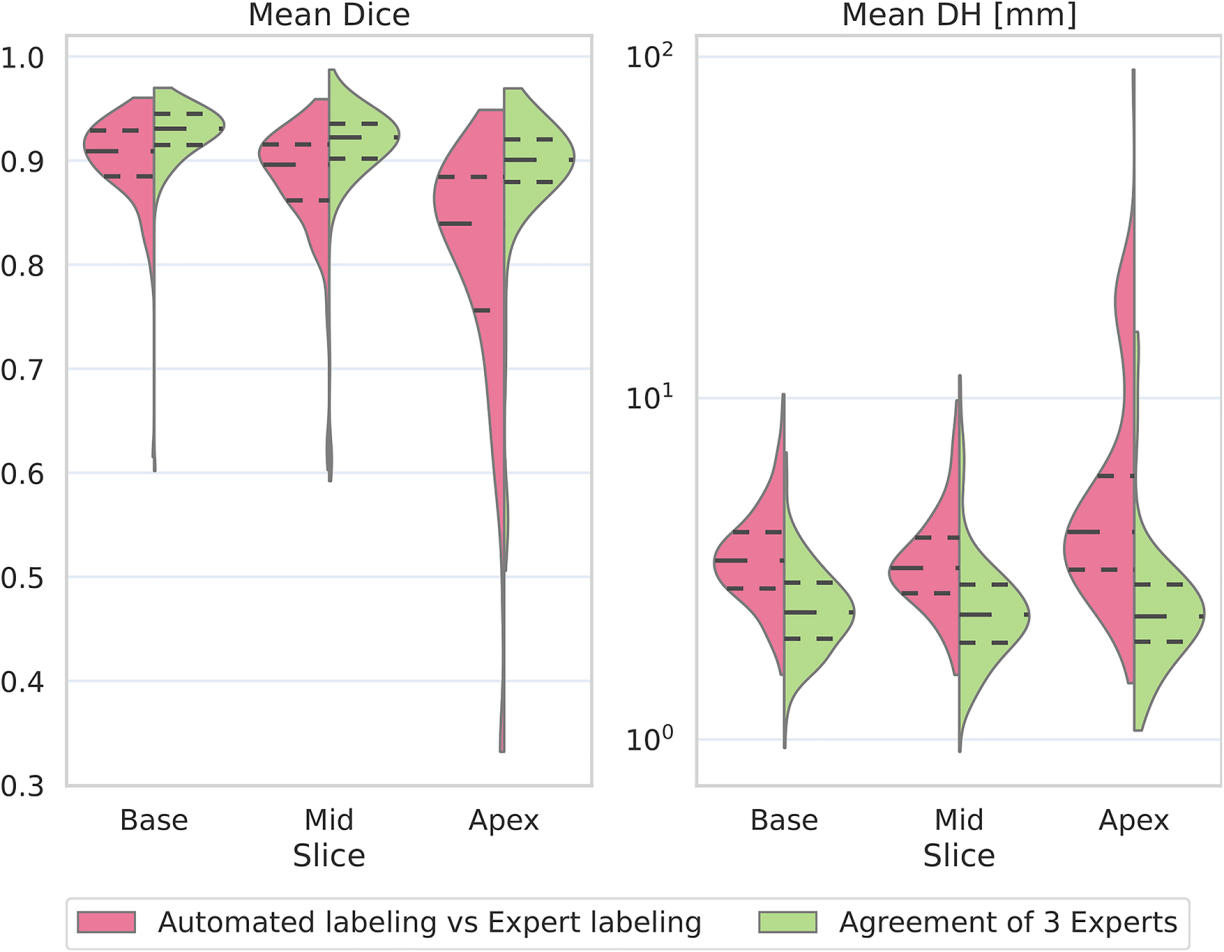
Per-slice violin plots comparing the agreement of our model with experts to agreement among experts. Table 1 shows the number of mapping images we used for training and evaluation (testing). Violin plots were generated on the test set.

Figure 8 and Table 4 shows results separately for the three slices used in this study, and Table 1 the number of training and test samples for each slice. Both the Dice score and Hausdorff distance distributions show that the proposed automated method performs comparable to how experts agree on basal and midventricular slices. However, the lower apical Dice score highlights that apical slices are more challenging even for experienced annotators. Also, our deep learning method shows a higher accuracy degradation on apex slices than experts do.

### 4.3. T1 and T2 mapping values

Parametric T1 and T2 mapping images allow medical practitioners to diagnose cardiac diseases based on quantitative measures. The primary practical aim of our research is to develop methods that automate the identification of epi- and endocardial contours and thereby simplify the process of measuring myocardial T1 and T2 values. To assess our method’s ability to aid T1 and T2 value measurement, we calculate myocardial T1 and T2 mapping values based on the contours predicted by our model and compared to those derived from expert-determined contours (Table 5). Our model’s median T1 and T2 values were 1041.73 ms (IQR: 110.53 ms) and 56.24 ms (IQR: 13.2 ms) respectively. These values were comparable to those obtained by each observer (median T1 and T2 values across all observers were 1026.41 and 50.96 ms respectively). The relatively close interquartile ranges (IQRs) suggest good overall agreement between our model and human observers, indicating the robustness of our method. This is further supported by median T1 values not differing significantly between our automated method and experts.

The intraclass correlation coefficient for the average ratings across observers was 0.90 and 0.81 for T1 and T2 mapping, respectively, indicating good reliability. The 95% confidence interval for the ICC was 0.87 to 0.93 for T1 and 0.75 to 0.86 for T2.

### 4.4. Ablation

#### 4.4.1. Network architecture

To investigate the effectiveness of our proposed network architecture, we compare our results to a U-Net that follows the original architecture proposed by Ronneberger et al. (22). Data for training and testing is identical to that used in the rest of our work. Table 6 shows that the proposed architecture (using a residual encoder and a lightweight decoder) outperforms the original U-Net architecture, while also offering faster training and inference times.

**Table 6:** Ablation study for the effect of the architecture on the segmentation performance.

	Original U-Net (22)	Resnet50 encoder with lightweight decoder (Ours)
Inference time for one slice (s)	0.42	0.18
Training time (min)	90	60
Number of parameters	31M	32M
IoU - Jaccard index ⇑ (%)	73.21	80.02
Dice score ⇑ (%)	79.14	86.39
Hausdorff distance ⇓ (mm)	15.4	4.69
Mean surface distance ⇓ (mm)	8.89	2.11

⇑ indicates higher-the-better metrics, while metrics ⇓ are lower-the-better.

#### 4.4.2. Pretrained encoder

We conducted experiments with initializing the encoder’s weights randomly and from a pretrained ImageNet model. Table 7 shows that initializing the encoder’s weights from an ImageNet pretrained encoder provides 5% in terms of Dice score over random initialization. All further inspected metrics were also superior for the pretrained approach.

**Table 7:** Ablation study for the effect of pretrained encoder on the segmentation performance.

	Without pretrained encoder	With pretrained encoder
IoU - Jaccard index ⇑ (%)	75.11	80.02
Dice score ⇑ (%)	81.69	86.39
Hausdorff distance ⇓ (mm)	9.63	4.69
Mean surface distance ⇓ (mm)	3.82	2.11

The decoder is always initialized randomly. ⇑ indicates higher-the-better metrics, while metrics ⇓ are lower-the-better.

#### 4.4.3. Post-processing

**Figure A1:**
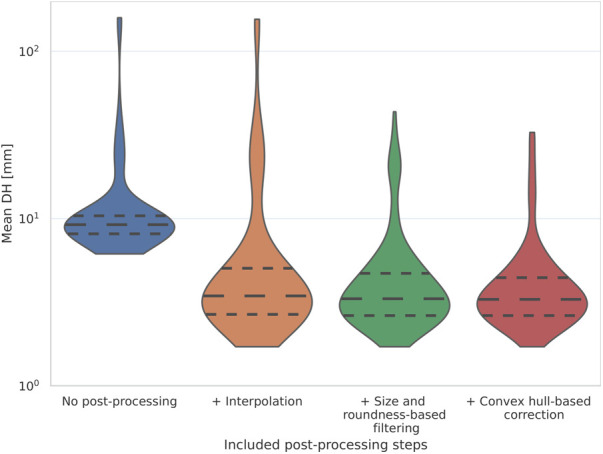
Ablation results showing the effect of each step in the proposed contour fitting pipeline. The figure shows the distribution of Hausdorff distance results over all samples for the same model, with the indicated post-processing steps added to the pipeline.

Ablation experiments are conducted to verify that each post-processing step we propose in Section 3.6 improves the accuracy of the fitted contours. Following the contour fitting algorithm (29) we add each post-processing step one-by-one and report the mean Hausdorff distance results of the same model in Table 8. Additionally, we include the distribution of Hausdorff distance results over all samples for each post-processing step in the Appendix, Figure A1. Since the Dice score is independent of the contour-fitting pipeline, we report only the Hausdorff distance results. We observe that each post-processing step improves the fitted contour accuracy. The most significant improvement is achieved by the interpolation step. In particular, this is due to the fact that we calculate the Hausdorff distance for the discrete points of the predicted and ground truth contours. Due to the interpolation, Hausdorff distance computations are considerably more accurate. In cases where the predicted segmentation map includes additional incorrectly labelled pixels, the fitted contours must be filtered to select the one that is most likely the correct one. This is accomplished through the filtering step, which results in a significant improvement in the Hausdorff distances. Finally, the correction step based on the convex hull of the predicted contour improves contours in a few cases and therefore improves the overall accuracy of the fitted contours.

**Table 8:** Ablation results showing the effect of each step in the proposed contour fitting pipeline.

	Hausdorff distance (mm)
No post-processing (only contour-fitting by (29))	14.67
+ Interpolation	9.91
+ Size- and roundness-based filtering	5.27
+ Convex hull-based correction	4.70

## 5. Discussion

This single center study based on 262 heterogeneous CMR cases may have important implications into clinical practice by providing high accuracy automatic segmentation for clinical use. Our algorithm can be implemented into the everyday clinical workflow after rigorous external validation. Automated contour prediction using our tool takes 12.3 sec for 100 slices on average, while this requires approx. 100 min for experts. The automated segmentation algorithm will contribute to the optimization and time efficiency of mapping post-processing, which is still unsolved by most commercially available tools. Our segmentation method provided reasonably good results for apical segments which are notoriously challenging. This heterogeneous data source will help maintain the stability of the results across CMR vendors and mapping sequences. Future research looking into the clinical validity of global mapping values derived from the implementation of this code will later permit automated segmentation of long axis images.

Our study’s novelty resides in three significant aspects. Firstly, we leveraged a U-Net based deep learning architecture, which, while widely used in cine CMR image segmentation tasks (6), has not been explored in T1 and T2 mapping segmentation. Our work optimizes the U-Net architecture for this specific application, along with tailored data processing techniques applied in the model training phase. Secondly, the segmentation of T1 and T2 mapping images presents unique challenges, as it contains more detailed information about tissue properties and, thus, slight discrepancies in segmentation can result in notably different measurements. Our study is one of the first to address these distinct challenges of mapping image segmentation (7). Finally, we augmented the robustness and generalizability of our segmentation tool by training and validating it on a diverse dataset comprising both clinical patients and healthy volunteers. This heterogeneous dataset ensures that our model is effective and applicable across various cardiovascular conditions.

Our tool holds significant implications for diagnosing and managing a variety of cardiovascular diseases by enabling faster quantitative analysis of critical diagnostic markers, potentially saving clinicians and researchers significant time and labor. Envisioned as an integral part of existing clinical workflows, our model’s key goal is to serve as an inline segmentation tool on CMR scanners. This integration would allow real-time analysis, facilitating a more streamlined diagnostic workflow, and enabling radiographers and physicians to adjust scanning protocols on-the-go if necessary, thus significantly enhancing patient care.

Our method shows very similar mid-ventricular and basal performance and the notably worse apical segmentation performance. We would like to emphasize multiple factors contributing to these results, as they contradict findings of previous studies reporting cine segmentation tools (6). First, our training dataset contains a lower number of labelled Apical slices (see Table 1). Second, the segmented region is the smallest on apical slices and the resolution of these areas is the worst, which makes this region challenging both for our model and experts. Third, in our center we take particular care during mapping slice planning, and we aim to ensure that the basal slice does not contain the aortic outflow tract. This planning approach reduces the complexity of the basal slices in our dataset, allowing our algorithm to perform more effectively in these regions. Finally, the blood-myocardium contrast in mapping images is inherently different from that in cine images, which are commonly used for most segmentation tool development. Our algorithm addresses the unique challenges posed by this contrast difference, demonstrating its ability to handle these characteristics and provide efficient, accurate segmentation.

The potential of our tool to be incorporated in clinical practice is also highlighted by our results on estimating T1 and T2 values values. Non-significant differences between T1 values obtained using our tool and those obtained by experts indicated that our model’s accuracy is comparable to that of experts on T1 mapping images.

Our automated model yielded a slightly elevated median T2 value of 56 ms, which can be attributed to multiple factors. Notably, our training and testing datasets encompassed a diverse set of patients with varying cardiac conditions, including myocarditis, post-heart transplant, and hypertrophic cardiomyopathy. Each of these conditions is known to potentially elevate T2 values due to the presence of myocardial edema or fibrosis. The borderline increased T2 values observed in both our training (median 52 ms, IQR 7 ms) and testing sets (median 51 ms, IQR) as provided by expert annotation confirms this assertion. It is important to clarify that our model was designed with an emphasis on segmentation accuracy, rather than specifically trained on predicting T2 values. Despite this, the input data, with its broad pathological range, may have indirectly influenced the resulting T2 values.

One of the challenges with automated models is the potential for segmentation errors. Even minor discrepancies in segmentation can significantly influence the derived T2 values. In our case, if the model tends towards over-segmentation, it might include areas affected by partial volume effect that is excluded by the expert readers, thereby inflating the computed T2 values. Our finding highlight the need for stringent quality control procedures when deploying automated tools in clinical scenarios. Such measures can help ensure segmentation accuracy, providing more reliable T2 mapping values. Furthermore, it underscores the necessity of ongoing research to understand these discrepancies better, to continuously refine our model (for example through further optimizing for mapping values), and to enhance its clinical utility in a diverse patient population.

This is a relatively small, single center study using CMR data from a single vendor and scanner, which prohibits the generalizability of our results. Of note all study participants are of Caucasian ancestry, therefore we could not test the fairness of our algorithm in terms of a more diverse ethnic background. Moreover, the dataset is biased in gender (∼71% male vs ∼29% women), which might also affect our outputs. To maximally preserve patient privacy, we removed all metadata, such as sex, age, ethnicity and body size measure, from our dataset. As a result, we did not analyze our method’s segmentation accuracy with respect to variables. We believe that different types of diseases (e.g., HCM, DCM) have a much greater influence on the efficiency of our segmentation model, however, to ensure optimal clinical implementation, future works should investigate the effect of these variables on the accuracy of our model.

Deep learning models could be improved in many cases by adding more data and building bigger models, according to the power law of deep learning (32). For much more general use, the inclusion of additional data (from other sites, scanners, field strength) would be essential. It is the theoretical best error, that can be reached by exploiting the information content of the data, namely T1 and T2 mapping images in this case. It is our belief that the consensus labeling is a good approximation of this region. Although the current discrepancy between the consensus label and the AI-based model is small, it may be further reduced by incorporating more data in the training phase.

**Figure 9:**
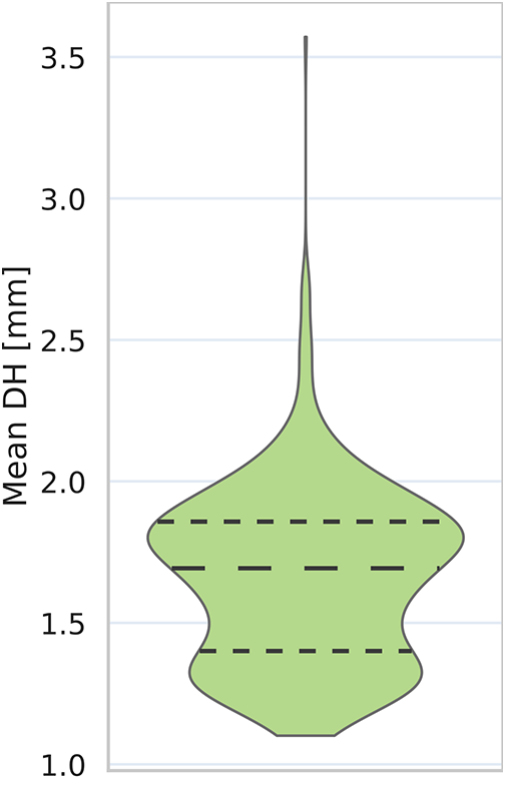
Distribution of achievable best Hausdorff distance values. We measure achievable lowest contour accuracy for each mapping by converting ground truth contours to mask, fitting contours to these, then computing Hausdorff distance between these and the initial ground truth contour.

Moreover, the limitation of the deep learning approach are the following:

∙ Anamnesis or any additional information describing the patients are not used by our segmentation method, therefore it cannot incorporate these into its predictions.
∙ Our approach is based on a simple 2D segmentation network, therefore it is unable to learn and reason based on 3D structure of the hearth.
∙ In this study we do not conduct confidence estimation, or apply an explainability method to the applied automated method.
∙ We ran experiments with a single model. Introducing other architectures (e.g. vision transformers (33)) and massive hyperparameter optimization (34) is the subject of future research.
∙ Producing segmentation masks from ground truth contours results in some information loss, because contours are given more accurately than the resolution of the segmentation masks. This leads to noticeable inaccuracy of contours fitted ground truth segmentation masks, which presents the best achievable accuracy for our model. We can measure this value for each mapping by converting ground truth contours to mask, fitting contours to these, then computing Hausdorff distance between these and the initial ground truth contour. The distribution of the best possible values is plotted on Figure 9.

## 6. Conclusion

In this study, we successfully developed and demonstrated the feasibility of a deep learning model for the segmentation of cardiac T1 and T2 mapping images. Our AI-based model, trained on over 7,000 raw mapping images from 262 participants, showed minimal discrepancies when compared with expert consensus, affirming its accuracy and reliability. The challenges encountered during the development of our model underlined the complexity of CMR T1 and T2 mapping segmentation. Our research underscored the need for future work to not only focus on achieving accurate segmentation, but also on ensuring the accurate measurement of tissue properties. The promising initial results from our model suggest potential for its integration directly into scanner systems. This prospect could significantly streamline clinical routines and enhance the diagnosis and management of cardiovascular diseases. Ultimately, our work represents a significant step towards the development of real-time, automated tools for CMR mapping analysis.

## Figures and Tables

**Figure A2:**
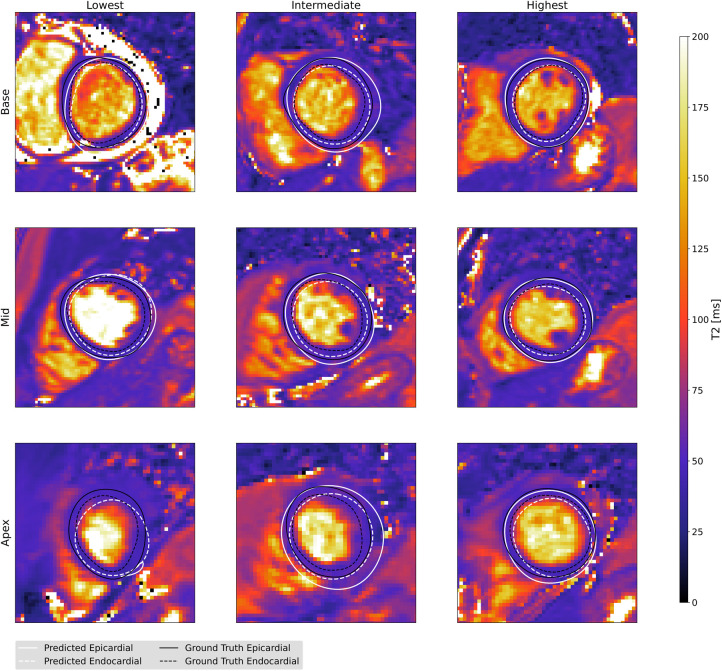
Predicted and ground truth contours with low, intermediate and high Hausdorff distance results, selected separately for apical, midventricular and basal T2 maping images.

**Figure A3:**
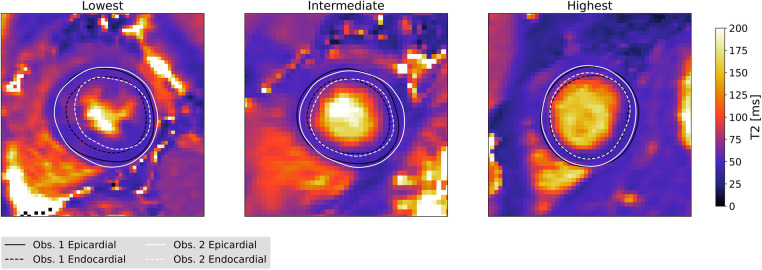
Example contours from two experts with different levels of agreement (measured by Hausdorff distance), plotted for two observers as comparison baseline to Figure A2. All subfigures show apical T2 maps.

## Data Availability

The source code can be accessed on the following location: https://github.com/BME-SmartLab/CardiacMappingSeg. Raw data are available upon request to the corresponding author.

## Footnotes

^1^https://github.com/qubvel/segmentation˙models.pytorch, access date: 10 November 2022.

^2^https://github.com/rwightman/pytorch-image-models, access date: 10 November 2022.

^3^https://opencv.org/, access date: 10 November 2022.

^4^https://albumentations.ai/, access date: 10 November 2022.

## Appendix

We supplement the results of Section 4.4.3 with Figure A1 showing the effect of each step in the proposed contour fitting pipeline. For this, we show the distribution of Hausdorff distance results over all samples for the same model, with the indicated post-processing steps added to the pipeline.

Figures A2 and A3 show qualitative results on T2 mapping images, comparing our model to one observer (Figure A2) and illustrating interobserver variability as a reference (Figure A3).

### Dedicated model for T1 and T2 mapping images

Training a dedicated model for T1 and T2 mapping images separately could have the benefit of better fitting to each type of image, as these have different intensity range and characteristics. To verify this hypothesis, we modify our methods to train and test dedicated models on T1 or T2 images only, while keeping all other hyperparameters unchanged. Table A1 shows the results of dedicated T1 and T2 models, evaluated on T1 and T2 images respectively, and includes the resuts of the combined model separately on T1 and T2 images (as in Table 3) for comparison. The results show that the dedicated models perform better than a combined model. The accuracy improvement of dedicated models on T2 mapping is statistically significant, however not on T1 mapping. In our opinion, these improvements are not guaranteed to transfer to larger more diverse (multi-center) datasets, and don’t justify the added complexity resulting from training, fine-tuning and deploying separate models. Therefore, we choose to use a single model for both T1 and T2 mapping images in our experiments.

**Table A1:** Ablation study comparing dedicated models for T1 and T2 mapping images with a combined model. The table shows the mean and standard deviation of each metric over all samples.

Model	Combined model		T1 only model	T2 only model
Test sample type	T1	T2	T1	T2
IoU - Jaccard index ⇑ (%)	81.7 (9.07)	78.39 (8.8)	82.41 (8.16)	82.73 (8.3)
Dice score ⇑ (%)	87.8 (8.79)	85.02 (9.12)	88.51 (7.51)	88.78 (8.14)
Hausdorff distance ⇓ (mm)	3.97 (3.36)	5.39 (7.69)	3.79 (3.65)	4.55 (9.41)
Mean surface distance ⇓ (mm)	1.77 (1.31)	2.44 (5.52)	1.77 (1.95)	2.21 (6.11)
